# Associations between Health Literacy, Trust, and COVID-19 Vaccine Hesitancy: The Case of Hong Kong

**DOI:** 10.3390/vaccines11030562

**Published:** 2023-03-01

**Authors:** Cindy Yue Tian, Phoenix Kit-Han Mo, Dong Dong, Hong Qiu, Annie Wai-Ling Cheung, Eliza Lai-Yi Wong

**Affiliations:** 1 JC School of Public Health and Primary Care, Faculty of Medicine, The Chinese University of Hong Kong, Hong Kong 999077, China; 2 Centre for Health Systems and Policy Research, JC School of Public Health and Primary Care, The Chinese University of Hong Kong, Hong Kong 999077, China

**Keywords:** health literacy, COVID-19, vaccine hesitancy, mediation analysis

## Abstract

This study investigates how health literacy (HL) and trust in health information affected COVID-19 vaccine hesitancy among Chinese Hong Kong adults. A cross-sectional study was conducted in August 2022. A total of 401 participants completed the study. Participants completed a newly developed Hong Kong HL scale and self-reported their trust levels in health information from different resources. The proportions of early uptake of the first dose and booster dose of COVID-19 vaccine were 69.1% and 71.8%, respectively. The risk of delaying the first dose was higher among participants with inadequate functional HL (OR = 0.58, *p* = 0.015), adequate levels of two subdomains of critical HL (OR = 1.82, *p* = 0.013; OR = 1.91, *p* < 0.01), and low-level trust in health information from the government (OR = 0.57, *p* = 0.019). Respondents with adequate interactive HL (OR = 0.52, *p* = 0.014) and inadequate level of one subdomain of critical HL (OR =1.71, *p* = 0.039) were more likely to delay the booster dose. This negative association between critical HL and vaccination was suppressed by trust in health information from the government. This study shows that HL and trust in health information from the government are associated with COVID-19 vaccine hesitancy. Efforts should be directed at providing tailored communication strategies with regard to people’s HL and increasing public confidence in health authorities to decrease vaccine hesitancy.

## 1. Introduction

Vaccine hesitancy (i.e., delay in acceptance or refusal of vaccines) has been a common phenomenon during the pandemic. It also may be a key factor in leading Hong Kong to the highest daily death per capita in the fifth wave that started in early Jan 2022 [1,2]. During this wave, almost half of Hong Kong residents were estimated to have been infected with COVID-19 [3], and around 73% of COVID-related deaths were unvaccinated [1]. Notably, when this wave hit this city, less than 67% of the population was vaccinated with the first, and only 6% was vaccinated with the booster dose [4]. Induced by the surge of confirmed cases and high death rates among unvaccinated people, the vaccination rates climbed; in early March, the proportion of those with first and booster doses increased to 88% and 32%, respectively [4]. Although this devastating wave has subsided, identifying the determinants of vaccine hesitancy is critical to boosting vaccination rates in future vaccine campaigns globally. 

One potential factor affecting vaccine hesitancy could be health literacy (HL), which refers to an individual’s ability to process and use health information to promote health [5]. Theoretically, people with sophisticated HL are more likely to understand health information and respond in a manner that benefits their health, especially for vaccination programs that involve complex and evolving information. However, two recent systematic reviews highlighted that there is limited evidence to support the association between HL and vaccine hesitancy, and this association remains unclear across vaccine types [6,7]. Similarly, although the relationship between HL and COVID-19 vaccination has been investigated, the results are inconsistent [8,9,10,11,12,13,14,15,16]. One study conducted among midwifery students indicated that the students’ decisions to receive the COVID-19 vaccination were not affected by their HL levels [8], while one Australian study argued that inadequate HL was significantly associated with reluctance towards COVID-19 vaccination [12]. Therefore, further studies are needed to enable a more precise picture of the impact of HL on COVID-19 vaccine hesitancy. 

Moreover, a comprehensive measurement of HL is needed to investigate the association between HL and vaccination. According to Nutbeam’s theory, HL is not just about functional health literacy (FHL), accessing and reading the information; it also involves interactive health literacy (IHL), in which cognitive and social skills are needed to comprehend information from different forms of communication. Then, critical health literacy (CHL), which refers to a higher level of cognitive and social skills, is required to critically analyze information and employ this information to gain better control over life events [17]. However, previous studies investigating this association between HL and vaccination mainly focused on FHL, which is a basic level of HL [6,7]. Additionally, a major challenge in the pandemic is how individuals can integrate and transfer the abundance of information into proper behaviors that not only affect them, but also their families and community. CHL might be the key in light of such a challenge. According to the latest understanding of CHL [18,19,20,21], CHL includes the ability to judge the quality of information (i.e., CHL-1), be aware of the social structural factors that influence health outcomes (i.e., CHL-2), and actively transform knowledge into action to address the modifiable determinants of health for personal and community health (i.e., CHL-3). Linked to the scenario of the COVID-19 vaccination, a critical health-literate citizen is expected to be able to question information from the internet and understand herd immunity as well as make informed decisions to get vaccinated for self-protection and public good. However, most of the studies mainly focused on individuals’ ability to judge the information (i.e., CHL-1) and did not capture all the components of CHL mentioned above [9,22,23,24]. This is the gap this study aimed to address.

Trust in health information (trust) has been identified as an essential factor associated with COVID-19 vaccination across countries [25,26,27]. Nevertheless, there is limited evidence [28,29,30] about how people intend to take COVID-19 vaccination considering their trust and HL levels. The Health Literacy Skills Framework highlights that greater levels of HL may lead to greater compliance with vaccine recommendations, and trust, as one potential mediator, may contribute to such engagement in vaccination programs [31]. Aligned with this, the present study aimed to provide empirical evidence of the Health Literacy Skills Framework by investigating how trust impacts vaccination uptake among people with inadequate HL. 

From all these perspectives, this study aimed to use a comprehensive HL scale to examine the associations between HL, trust, and COVID-19 vaccine hesitancy in Hong Kong. We hypothesized that low HL is directly associated with delayed COVID-19 vaccine uptake, and trust may mediate this association.

## 2. Methods

### 2.1. Study Design and Patient Participation

A cross-sectional study was performed between August to September 2022. Participants who were aged 18 years or older and permanent Hong Kong residents who could read Chinese were recruited from the registrants of an internet research service company called Qualtrics. The participants were invited to complete the survey by email and message. If they accepted, they could click the survey link to fill out the online questionnaire. All participants provided informed electronic consent before participation. According to a systematic review of the prevalence of inadequate HL in Southeast Asian countries (range: 1.6–99.5%; mean: 55.3%) [32], we took 55.3% as the expected prevalence of inadequate HL in Hong Kong. With consideration of a 95% confidence level and 5% allowable error, the estimated adequate sample size should be 380 or above [30]. 

Given that online surveys may be biased towards young people who may possess better digital literacy, we used quota sampling with consideration of age. We also took the distributions of gender and living district into account to reach a regionally representative sample. Hence, a quota sampling was conducted to match the distribution of participants by gender (i.e., female and male), age group (i.e., 18–24, 25–34, 35–44, 45–54, and ≥55 years), and living district (i.e., Hong Kong Island, Kowloon, and New Territories) to the results of the 2020 Hong Kong census [33]. We piloted the survey among 30 participants and found that the median time for them to complete the survey was 9 min. Based on the results, we added half of the median completion time (i.e., 270 s) as the speeding check to terminate those who did not respond thoughtfully. We also added two attention-check questions to ensure that participants were reading each question carefully. If participants failed the two questions, their survey would be terminated as well. As far as the survey went, we monitored who took the survey and the number of participants for each quota sample. This survey stopped when the quota for each stratum was met.

### 2.2. Measures

#### 2.2.1. Study Variables

**Figure 1:**
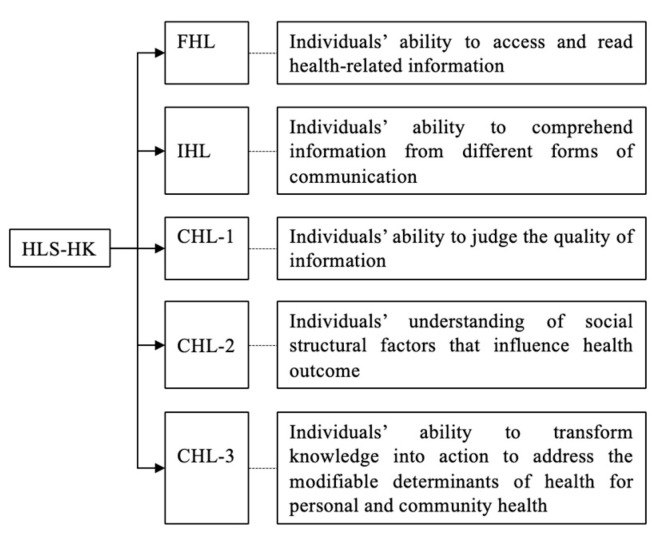
Theoretical framework of HLS-HK. (Note: Abbreviations: HL: health literacy; FHL: functional health literacy; IHL: interactive health literacy; CHL-1: the first subdomain of critical health literacy; CHL-2: the second subdomain of critical health literacy; CHL-3: the third subdomain of critical health literacy; HLS-HK: Hong Kong Health Literacy Scale).

Participants’ HL was measured using the Hong Kong Health Literacy Scale (HLS-HK) developed and validated by our research team. The scale comprehensively operationalized the five domains mentioned above: FHL, IHL, CHL-1, CHL-2, and CHL-3 (see Figure 1). Its content validity was examined by local healthcare providers and consumers in one Delphi study [34]. Furthermore, its internal consistency, factorial structural validity, convergent validity, and predictive validity were assessed in one cross-sectional survey among 433 Hong Kong Chinese [35]. The results demonstrate overall good psychometric qualities of the scale in the context of Hong Kong [34,35]. The process of the scale development and validation has been documented elsewhere [34,35], and the present study was the first to explore the association between HL and other variables in Hong Kong using the scale after its development. In the present study, Cronbach’s alpha of the total scale was 0.90. The confirmatory factor analysis shows an acceptable fit of the five-domain framework, with a comparative fit index (CFI) = 0.90, standardized root mean square residual (SRMA) = 0.07, and root mean square error of approximation (RMSEA) = 0.06. 

All items were rated on a 5-point Likert scale, and the scores were summed. Participants who scored a higher score on this scale had a higher level of HL. The mean values of the individual domains of HL were used to divide the sample into inadequate vs. adequate groups. Additionally, given that participant HL was measured after the fifth wave and their HL might evolve within health education programs during this wave, we conducted propensity score matching (PSM) to examine whether subjects’ HL were relatively stable over time. Based on age, gender, income, education, and self-reported health status, participants in our previous survey (conducted before and in the fifth wave) [35] and the current survey (conducted after the fifth wave) were propensity score matched at a 1:1 ratio. The results show no difference in the HL levels between the two groups (see Tables S1 and S2 in the supplement). Therefore, it is reasonable to assume that the participants’ HL did not change substantively across the fifth wave. 

Participants’ trust levels were measured by items adopted from previous studies [36,37]. They were asked about their perceived trust in health information from the government, healthcare professionals, family members and friends, social media (e.g., Facebook, Instagram), and mass media (e.g., newspapers, magazines) by rating a 5-point Likert scale (1 = distrust completely to 5 = trust completely). The responses were then grouped into “distrust” (scored 1–3) and “trust” (scored 4–5). 

#### 2.2.2. Study Outcomes

Participants were asked about their COVID-19 vaccination records. Given the low vaccine coverage rate before the fifth wave, we used the start date (1 January 2022) of this wave to dichotomize individuals into two groups: early and late first dose vaccinees. Considering the vaccination schedule (i.e., the earlier to receive the first dose vaccine, the earlier to be fully vaccinated), we also used booster dose uptake to indicate vaccine hesitancy. Namely, those who received the booster dose and those who did not receive the booster dose during the date of the survey were categorized as early and late booster dose vaccinees, respectively. 

#### 2.2.3. Sociodemographic and Health-Related Characteristics 

Age, gender, educational attainment, monthly household income, employment, marital status, health status, chronic condition, and health behaviors related to smoking, physical activity, and alcohol were self-reported and collected and then grouped as dichotomous variables. 

### 2.3. Statistical Analysis

Descriptive statistics with proportions were calculated. Sociodemographic and health-related variables, HL and trust were stratified by individuals’ COVID-19 vaccination uptake, and Chi-square tests were performed to assess variation across the categories. Multivariable binary logistic regression using the forward procedure was performed to examine the effects of HL and trust on individuals’ COVID-19 vaccine hesitancy. The first multivariable model (Model 1) tested the association between the individual domains of HL and vaccine hesitancy. The second multivariable model (Model 2) examined the impact of the individual domains of HL and trust on vaccine hesitancy. Age, gender, educational attainment, income, health status, and chronic disease status were the covariates in the two models. Sensitivity analyses using the original categorizations for Likert questions were performed to ensure the robustness of the results. Additionally, according to Baron and Keeny’s method for mediation [38], we only included variables with statistical significance in Model 1 and Model 2 in the mediation models to test any potential indirect effects of trust on the association between HL and delayed vaccination. All data analyses were conducted using SPSS version 26 [39] and R software (MatchIt package [40] and mediation package [41]). *p* values of 0.05 were used to determine statistical significance. 

## 3. Results

A total of 401 participants completed the survey. Table 1 presents the sociodemographic and health-related characteristics of the participants. The sample distribution in terms of age, gender, and living district was almost in accordance with the distribution of these metrics in the Hong Kong population (see Table S3 in the Supplementary Materials) [33]. The proportions of early first and booster dose vaccinees were 69.1% and 71.8%, respectively. 

### 3.1. Health Literacy

Overall, most of the participants had insufficient HL (see Table 2). Specifically, over half of the participants had inadequate CHL-1 (55.9%) and CHL-3 (59.9%), and nearly half of them had inadequate FHL (44.4%), IHL (42.9%), and CHL-2 (46.6%). FHL (*p* = 0.030), IHL (*p* = 0.014), and CHL-3 (*p* = 0.004) were significantly associated with first dose vaccine hesitancy. The differences between IHL (*p* = 0.002), CHL-1 (*p* = 0.041), CHL-3 (*p* = 0.029) and booster dose vaccine hesitancy were significant. 

### 3.2. Trust

Table 2 indicates that the proportion of trust in health information from healthcare professionals (69.6%) was the highest, followed by government (45.9%), family members and friends (39.9%), mass media (27.2%) and social media (15.7%). Although there was no significant difference between trust and vaccine hesitancy, trust in information from the government (early vs. late first dose: 48.0% vs. 41.1%) and healthcare professionals (early vs. late booster dose: 71.5% vs. 65.3%) tended to reduce vaccine hesitancy.

### 3.3. Health Literacy, Trust, and COVID-19 Vaccine Hesitancy

In multivariable analysis (see Table 3), Model 1 indicated that respondents with a higher level of FHL were more likely to receive an early first dose vaccine (OR = 0.56, 95% CI = 0.36–0.88, *p* < 0.05). Contrarily, people with an adequate level of CHL-1 (OR = 1.63, 95% CI = 1.03–2.58, *p* < 0.05) or CHL-3 (OR = 1.67, 95% CI = 1.06–2.63, *p* < 0.05) were more likely to receive their first dose vaccine late. For the booster dose hesitancy, the risk of delaying this dose was significantly higher among participants with inadequate IHL (OR = 0.51, 95% CI = 0.33–0.81, *p* < 0.01) and adequate CHL-3 (OR = 1.82, 95% CI = 1.15–2.87, *p* < 0.05). 

With the inclusion of trust in Model 2, the effect of FHL, CHL-1, and CHL-3 on the first dose vaccine and the effect of IHL and CHL-3 on the booster dose remained strong. Trust in information from the government was significantly positively associated with first dose vaccine hesitancy (OR = 0.57, 95% CI = 0.35–0.91, *p* < 0.05). However, there were no significant associations between trust in health information from other resources and vaccine hesitancy. The sensitive analysis confirmed these findings (see Table S4 in the Supplementary Materials).

### 3.4. Mediation Effect of Trust on Health Literacy and First Dose COVID-19 Vaccine Uptake

Based on the results of the above multivariable analysis, we only tested the potential mediation effects of trust in health information from the government on the association between certain domains of HL (i.e., FHL, CHL-1, CHL-3) and first dose vaccine hesitancy after controlling all other variables. Table 4 indicates that the effect of CHL on first dose vaccine hesitancy was significantly suppressed by trust in information from the government (CHL-1-> trust in health information from the government -> first dose vaccine hesitancy: standardized indirect effects = −0.012 < 0, 95%CI = −0.028–0.00, *p* = 0.040; CHL_3 -> trust in health information from the government -> first dose vaccine hesitancy: standardized indirect effects = −0.016 < 0, 95% CI = −0.033–0.00, *p* = 0.014). There was no indirect effect of trust in information from the government on the association between FHL and first dose vaccination. 

## 4. Discussion 

### 4.1. Main Results

Decisions to vaccinate are complex, requiring an understanding of the scientific evidence and the adverse events that may occur during immunization schedules. HL is an important factor affecting this decision making. In this study, we used HLS-HK, which has shown good reliability and validity in measuring HL in a standardized process of scale development and validation. Although this is the first time this scale was adopted to explore the impact of HL on vaccine hesitancy, it provided important insights into the barriers and facilitators affecting vaccine uptake via comprehensively measuring FHL, IHL, and CHL. Specially, the multivariable analysis indicates that the basic acquisition of information at the FHL level is positively associated with the early first uptake of vaccination. This result is consistent with other studies [42,43,44]. It is reasonable to assume that people who were better at searching and understanding health-related information at the early stage of the pandemic were more likely to actively get vaccinated. Regarding IHL, participants with sufficient IHL were less likely to delay the booster dose. It seems that good communication with healthcare professionals could help increase immunization levels and reduce withdrawal from vaccination campaigns. However, higher levels of CHL are negatively associated with first and booster doses. This negative association is contrary to the expectation that people with high HL adopt more positive health behaviors. This behavior, however, has been documented in several other studies [45,46,47]. There are some potential explanations for this result. First, people with lower CHL might be less concerned about the effectiveness and side effects of the vaccines [46]. Thus, such people might be less hesitant to take the vaccine. Second, according to the theory of confirmation bias, when people who distrust vaccination also have higher HL, they are even more likely to choose the information that matches their biases and supports their beliefs [47]. These skeptics are unlikely to be reassured by healthcare authorities to get vaccinated. In these complex situations, health practitioners may need to listen to public concerns and facilitate targeted communication strategies to improve vaccine coverage. 

Additionally, this study revealed that over half of the participants were found to have a low score of HL. Those people were grouped as inadequate HL groups in our study. Other similar studies may group those people as insufficient or limited or low HL groups [32,48,49,50,51]. As these and our surveys revealed, the poor state of HL is a public health problem that is common across the globe. For example, one survey conducted among 8698 Chinese found that around 80% of subjects have inadequate HL [50]; another survey in Turkey found that 81.5% of individuals with diabetes have an inadequate level of HL [51]. Therefore, health education is needed to improve public HL and ensure equal access to health information not only physically but also literally. 

Although the information from the government was not the most trustworthy for the respondents in our study, it significantly affected their vaccination uptake. This study highlights that individuals with a higher level of trust in information from the government were more likely to receive the first dose vaccine early. This result is similar to several local studies [52,53,54] and reveals that people learn about the pandemic and take action to prevent infection not only considering the content and quality of the information but also their levels of trust in information from their government [25,55]. We also found that the standardized indirect/mediated effects of trust in information from the government on the associations between two subdomains of CHL and first dose vaccination were negative. This means the direct pathway (i.e., higher CHL -> later vaccination) was counteracted by the indirect pathway (i.e., higher CHL -> more trust -> earlier vaccination); as a result, the total effect of CHL on vaccination became smaller because the direct and indirect effects cancelled each other out. Therefore, trust in health information from the government served as a possible mechanism for suppressing this negative association between CHL and first dose vaccination hesitancy. Namely, as individuals with higher levels of CHL were less likely to engage with the vaccination protocol, building trust may be helpful to mitigate and eliminate vaccine hesitancy among these people. This may be because a higher level of trust in health authorities may repress the spread of vaccine conspiracy theories and consequently increase vaccine coverage rates [56,57].

### 4.2. Implications

This study indicates that different aspects of HL have different influences on vaccination hesitancy. Given the positive association between FHL and first dose vaccination and IHL and booster dose vaccination, healthcare practitioners may need to ensure that the public is given equal access to health information in the early stage of vaccine campaigns and use effective communication channels to remind people to adhere to clinical recommendations after they complete a primary series of vaccines. Regarding the negative association between CHL and vaccination, disclosing transparent information about the development and features of vaccination is the key to improve vaccine coverage rates [58,59]. In this way, people can critically analyze clear and unbiased information and thereby potentially avoid vaccine hesitancy. Moreover, considering that public trust in health information from the government might suppress the negative association between CHL and vaccine uptake, the government and health authorities may need to build, rebuild, or maintain trustful relationships with the public in future vaccine protocols.

### 4.3. Strengths and Limitations

A major strength of this study is the use of a comprehensive HL scale. Different standardized measurements [23,44,60,61,62,63] were adopted in previous studies investigating the relationship between HL and vaccination uptake. Unfortunately, they do not capture all related skills of HL, especially the skills related to the aspect of CHL. In this study, we used HLS-HK to address more dimensions of HL and added empirical evidence on the association between HL and vaccine hesitancy across immunization schedules. We also provided insights into how CHL may cause people’s under-reacting to vaccination during the pandemic.

However, this study has some limitations. First, all data collected were self-reported and recall biases might exist. Second, it might be possible that some aspects of HL were not measured in the HLS-HK due to the complexity of this concept. Third, the psychometric properties of HLS-HK are well proven but it is the first time this scale has been applied in a study, so the generalization of results may need more evidence to be supported. In addition, the validity of the HLS-HK across populations and contexts needs to be examined in future studies. Fourth, due to limited resources, we did not include a performance-based measure as a comparison scale to examine participants’ HL levels. Participants may overestimate their HL when they complete this self-reported scale. This may cause self-efficacy (i.e., an individual’s perception of their ability to get vaccinated) to confound the relationship between HL and vaccine uptake. Usually, those who overestimated their HL levels may tend to have a higher level of self-efficacy; people with sufficient self-efficacy are more likely to engage in vaccine campaigns. Fifth, selection biases might exist. Although we used quota sampling to reach a regionally representative sample, the study subjects, who were recruited from an online questionnaire platform panel, may be better at seeking and understanding information. Sixth, no causal relationship could be inferred by this cross-sectional study.

## 5. Conclusions

In this study, HL and trust in health information from the government are important factors affecting vaccine hesitancy. By using the newly developed scale HLS-HK to measure HL, we found that FHL, IHL and trust in information from the government are positively associated with COVID-19 vaccination. However, a negative association between CHL and this vaccine uptake was reported. These findings indicate a challenge for public health practitioners to effectively deliver health messages about vaccination to the general public, especially for those with inadequate FHL and IHL and adequate CHL. Efforts should be directed at providing tailored communication strategies about people’s HL and increasing public confidence in health authorities to raise vaccine coverage rates. In addition, given that the validity of HLS-HK across populations and countries is required, more studies are in demand to extend the implication of the results of this study to a broader context.

## Figures and Tables

**Table 1:** Sociodemographic characteristics and health-related factors of participants ^#^.

Variables	Total (*n* = 401)	First Dose			Booster Dose		
		Early (*n* = 277)	Late (*n* = 124)	*p* Value	Early (*n* = 288)	Late (*n* = 113)	*p* Value
Gender				0.107			0.615
Male	189 (47.1)	138 (49.8)	51(41.1)		138 (47.9)	51 (45.1)	
Female	212 (52.9)	139 (50.2)	73(58.9)		150 (52.1)	62 (54.9)	
Age groups				0.677			0.621
18–54	255 (63.6)	178 (64.3)	77 (62.1)		181 (62.8)	74 (65.5)	
≥55	146 (36.4)	99 (35.7)	47 (37.9)		107 (37.2)	39 (34.5)	
District				0.491			0.994
HK Island	70 (17.5)	45(16.2)	25 (20.2)		50 (17.4)	20 (17.7)	
Kowloon	120 (29.9)	87 (31.4)	33 (26.6)		86 (29.9)	34 (30.1)	
New Territories	211 (52.6)	145 (52.3)	66 (53.2)		152 (52.8)	59 (52.2)	
Education attainment				0.993			0.522
Secondary and below	123 (30.7)	85 (30.7)	38 (30.6)		91 (31.6)	32 (28.3)	
Post-secondary	278 (69.3)	192 (67.0)	86 (69.4)		197 (68.4)	81 (71.7)	
Employment status				0.980			0.683
Non-full-time	87 (21.7)	60 (21.7)	27 (21.8)		64 (22.2)	23 (20.4)	
Full-time	314 (78.3)	217 (78.3)	97 (78.2)		224 (77.8)	90 (79.6)	
Monthly household income				0.775			0.002 **
<40,000 HKD	193 (48.1)	132(47.7)	61 (49.2)		125 (43.4)	68 (60.2)	
≥40,000 HKD	208 (51.9)	145 (52.3)	63 (50.8)		163 (56.6)	45 (39.8)	
Marital status				0.945			0.042 *
Single ^a^	153 (38.2)	106 (38.3)	47 (37.9)		101 (35.1)	52 (46.0)	
Married	248 (61.8)	171 (61.7)	77 (62.1)		187 (64.9)	61 (54.0)	
Self-rated health status				0.871			0.746
Poor ^b^	190 (47.4)	132 (47.7)	58 (46.8)		135 (46.9)	55 (48.7)	
Good ^c^	211 (52.6)	145 (52.3)	66 (53.2)		153 (53.1)	58 (51.3)	
Chronic disease				0.228			0.243
Yes ^d^	120 (29.9)	88 (31.8)	32 (25.8)		91 (31.6)	29 (25.7)	
No	281 (70.1)	189 (68.2)	92 (74.2)		197 (68.4)	84 (74.3)	
Physical activities				0.660			0.976
Low ^e^	319 (79.6)	222 (80.1)	97 (78.2)		229 (79.5)	90 (79.6)	
High ^f^	82 (20.4)	55 (19.9)	27 (21.8)		59 (20.5)	23 (20.4)	
Smoking				0.869			0.189
Yes ^g^	37 (9.2)	26 (9.4)	11 (8.9)		30 (10.4)	7 (6.2)	
No ^h^	364 (90.8)	251 (90.6)	113 (91.1)		258 (89.6)	106 (93.8)	
Drinking				0.238			0.446
Yes ^i^	225 (56.1)	150 (54.2)	75 (60.5)		165 (57.3)	60 (53.1)	
No ^j^	176 (43.9)	127 (45.8)	49 (39.5)		123 (42.7)	53 (46.9)	

(Note: ^#^: Data are presented as the number of the participants in each category (*n*), together with the column percentage (%). *p*-values are obtained from the Chi-square test. a: single/widow/divorced/separated; b: individual’s self-report health status was poor/fair; c: individual’s self-report health status was good/very good/excellent; d: have at least one chronic disease; e: five days or over, at least 60 min vigorous or moderate activities or walking; f: not meeting the criteria for the “High” group; g: smoker; h: never smoke and former smokers; i: consuming alcoholic drinks during the last year; j: consuming zero alcoholic drinks during the last year; *: *p* < 0.05; **: *p* < 0.01).

**Table 2:** Bivariate relationships between COVID-19 vaccine hesitancy and HL and trust ^#^.

	Total (*n* = 401)	First Dose			Booster Dose		
		Early (*n* = 277)	Late (*n* = 124)	*p* Value	Early (*n* = 288)	Late (*n* = 113)	*p* Value
Adequate levels of five individual domain of HL:							
FHL	223 (55.6)	164 (59.2)	59 (47.6)	0.030 *	164 (56.9)	59 (52.2)	0.391
IHL	229 (57.1)	166 (59.6)	63 (50.8)	0.088	178 (61.8)	51 (45.1)	0.002 **
CHL-1	177 (44.1)	111 (40.1)	66 (53.2)	0.014 *	118 (41.0)	59 (52.2)	0.041 *
CHL-2	214 (53.4)	143 (51.6)	71 (57.3)	0.296	147 (51.0)	67 (59.3)	0.136
CHL-3	161 (40.1)	98 (35.4)	63 (50.8)	0.004 **	106 (36.8)	55 (48.7)	0.029 *
Trust in health information from:							
Government	184 (45.9)	133 (48.0)	51 (41.1)	0.201	140 (48.6)	44 (38.9)	0.080
Healthcare professionals	279 (69.6)	198 (71.5)	81 (65.3)	0.215	207 (71.9)	72 (63.7)	0.110
Family members and friends	160 (39.9)	103 (37.2)	57 (46.0)	0.097	110 (38.2)	50 (44.2)	0.265
Social media	63 (15.7)	40 (14.1)	23 (18.5)	0.296	45 (15.6)	18 (15.9)	0.940
Mass media	109 (27.2)	73 (26.4)	36 (29.0)	0.577	79 (27.4)	30 (26.5)	0.858

(Note: ^#^: data are presented as the number of the participants in each category (*n*), together with the column percentage (%). *p*-values are obtained from the Chi-square test; *: *p* < 0.05; **: *p* < 0.01. Abbreviations: HL: health literacy; FHL: functional health literacy; IHL: interactive health literacy; CHL-1: the first subdomain of critical health literacy; CHL-2: the second subdomain of critical health literacy; CHL-3: the third subdomain of critical health literacy).

**Table 3:** The associations among HL, trust, and COVID-19 vaccine hesitancy estimated from the multivariable logistic regression model ^a^.

Variables	First Dose		Booster Dose	
	Model 1	Model 2 ^b^	Model 1	Model 2 ^b^
	Adjusted OR (95% CI)	Adjusted OR (95% CI)	Adjusted OR (95% CI)	Adjusted OR (95% CI)
FHL	0.56 (0.36–0.88) *	0.58 (0.37–0.90) *	NS	NS
IHL	NS	NS	0.51 (0.33–0.81) **	0.51 (0.33–0.81) **
CHL-1	1.63 (1.03–2.58) *	1.82 (1.14–2.92) *	NS	NS
CHL-2	NS	NS	NS	NS
CHL-3	1.67 (1.06–2.63) *	1.91 (1.20–3.06) **	1.82 (1.15–2.87) *	1.82 (1.15–2.87) *
Trust in health information from:				
Government	/	0.57 (0.35–0.91) *	/	NS
Healthcare professionals	/	NS	/	NS
Family members and friends	/	NS	/	NS
Social media	/	NS	/	NS
Mass media	/	NS	/	NS

(Note: a: covariates included age, gender, educational attainment, income, health status, and chronic disease status; b: with the inclusion of trust in health information from the government, healthcare professionals, family members and friends, social media, and mass media; *: *p* < 0.05; **: *p* < 0.01; / not applicable. Abbreviations: HL: health literacy; FHL: functional health literacy; IHL: interactive health literacy; CHL-1: the first subdomain of critical health literacy; CHL-2: the second subdomain of critical health literacy; CHL-3: the third subdomain of critical health literacy; NS: not significant).

**Table 4:** Standardized direct, indirect, and total effects for the mediation model ^a^.

	Standardized Estimated Effects (95% CI)	*p* Value
FHL → trust in health information from the government → first dose vaccine hesitancy		
Indirect effects	0.001 (−0.017–0.02)	0.898
Direct effects	−0.104 (−0.215–0.01)	0.086
Total effects	−0.103 (−0.22–0.01)	0.084
CHL_1→ trust in health information from the government → first dose vaccine hesitancy		
Indirect effects	−0.012 (−0.028–0.00)	0.040 *
Direct effects	0.091 (0.006–0.15)	0.032 *
Total effects	0.079 (−0.007–0.14)	0.068
CHL_3 → trust in information from the government → first dose vaccine hesitancy		
Indirect effects	−0.016 (-0.033–0.00)	0.014 *
Direct effects	0.105 (0.035–0.15)	0.010 *
Total effects	0.089 (0.016–0.14)	0.020 *

(Note: a: bootstrapped confidence intervals were constructed using 1000 resamples; *: *p* < 0.05).

## Data Availability

The datasets generated and/or analyzed during the current study are not publicly available to protect the anonymity of participants but are available from the corresponding author on reasonable request.

## Supplementary Materials

The following supporting information can be downloaded at: https://www.mdpi.com/article/10.3390/vaccines11030562/s1, Table S1: Sociodemographic characteristics and health-related factors of original population and matched population; Table S2: Differences in health literacy among the matched population; Table S3: Participants’ sociodemographic characteristics compared with Hong Kong population; Table S4: The associations among HL, trust, and COVID-19 vaccine hesitancy estimated from the multivariable logistic regression model using the original categorizations for Likert questions.
