# Supplementary material for: Associations between Health Literacy, Trust, and COVID-19 Vaccine Hesitancy: The Case of Hong Kong
Source: Vaccines (Basel). 2023 Mar 1;11(3):562. doi: 10.3390/vaccines11030562 (PMC10059763; doi:10.3390/vaccines11030562)
Supplement: Supplementary file 1 [file vaccines-11-00562-s001.zip › vaccines-2194325-supplementary.pdf]

Table S1. Original Population and Matched Population

	Before PSM			After PSM		
	Current survey N (%)	Previous survey N (%)	P value	Current survey N (%)	Previous survey N (%)	P value
Total participants	401	433		392	392	
Gender			0.389			0.617
Male	189 (47.1)	217 (50.1)		185 (47.2)	192 (49.0)	
Female	212 (52.9)	216 (49.9)		207 (52.8)	200 (51.0)	
Age groups			0.457			0.442
18-24	42 (10.5)	53 (12.2)		40 (10.2)	46 (11.7)	
25-34	67 (16.7)	80 (18.5)		65 (16.6)	72 (18.4)	
35-44	77 (19.2)	70 (16.2)		77 (19.6)	62 (15.8)	
45-54	69 (17.2)	87 (20.1)		67 (17.1)	79 (20.2)	
≥ 55	146 (36.4)	143 (33.1)		143 (36.5)	133 (33.9)	
Education attainment			0.723			0.59
Primary and below	6 (1.5)	10 (2.3)		5 (1.3)	9 (2.3)	
Secondary	117 (29.2)	118 (27.3)		116 (29.6)	104 (26.5)	
Post-secondary (non-degree course)	63 (15.7)	75 (17.3)		63 (16.1)	65 (16.6)	
Post-secondary (degree course)	215 (53.6)	230 (53.1)		208 (53.1)	214 (54.6)	
Self-rated health status			0.888			0.916
Poor	27 (6.7)	26 (6.0)		27 (6.9)	25 (6.4)	
Fair	163 (40.6)	172 (39.7)		161 (41.1)	160 (40.8)	
Good	126 (31.4)	142 (32.8)		125 (31.9)	131 (33.4)	
Very good	71 (17.7)	82 (18.9)		66 (16.8)	67 (17.1)	
Excellent	14 (3.5)	11 (2.5)		13 (3.3)	9 (2.3)	

Note: PSM = propensity score matching

Table S2. Difference in health literacy among the matched population

	Mean (SD)	P value
Current survey	92.41 (13.10)	0.098
Previous survey	93.92 (12.29)	

Table S3. Participants' sociodemographic characteristics compared with Hong Kong population

Variables	Total (n =401)		2020 Hong Kong census
	n	%	%
Gender			
Male	189	47.1	44.9
Female	212	52.9	55.1
Age groups			
18-24	42	10.5	9.9
25-34	67	16.7	15.6
35-44	77	19.2	17.8
45-54	69	17.2	17.2
≥ 55	146	36.4	39.5
District			
HK island	70	17.3	16.6
Kowloon	120	29.9	30.6
New Territories	211	52.4	52.8

Table S4. The associations among HL, Trust, and COVID-19 vaccine hesitancy estimated from the multivariable logistic regression model using the original categorizations for Likert questions [a]

Variables	First dose		Booster dose	
	Model 1	Model 2 [b]	Model 1	Model 2 [b]
	OR(95% CI)	OR(95% CI)	OR(95% CI)	OR(95% CI)
FHL	0.56(0.36-0.88)*	0.60(0.38-0.94)*	NS	NS
IHL	NS	NS	0.47(0.30-0.74)*	0.47(0.30-0.74)*
CHL-1	1.63(1.03-2.58)*	1.75(1.10-2.80)*	NS	NS
CHL-2	NS	NS	1.79(1.14-2.81)*	1.79(1.14-2.81)*
CHL-3	1.67(1.06-2.63)*	1.87(1.18-2.99)**	NS	NS
Trust in health information from:				
Government	/	0.78(0.62-9,97)*	/	NS
Healthcare professionals	/	NS	/	NS
Family members and friends	/	NS	/	NS
Social media	/	NS	/	NS
Mass media	/	NS	/	NS

(Note: a = covariates included age, gender, educational attainment, income, health status, and chronic disease status; b = with the inclusion of Trust in health information from the government, healthcare professionals, family members and friends, social media, and mass media; * = $p < 0.05$; ** = $p < 0.01$; / = not applicable; Abbreviation: HL = health literacy; FHL = functional health literacy; IHL = interactive health literacy; CHL-1 = the first subdomain of critical health literacy; CHL-2 = the second subdomain of critical health literacy; CHL-3 = the third subdomain of critical health literacy; NS= not significant)
